# Misbeliefs about non-specific low back pain and attitudes towards treatment by primary care providers in Spain: a qualitative study

**DOI:** 10.1186/s12875-021-01617-3

**Published:** 2022-01-14

**Authors:** Ester García-Martínez, Jorge Soler-González, Joan Blanco-Blanco, Francesc Rubí-Carnacea, María Masbernat-Almenara, Fran Valenzuela-Pascual

**Affiliations:** 1 Department of Nursing and Physiotherapy, The University of Lleida, Montserrat Roig, 2, 25198 Lleida, Spain; 2 Group for the Study of Society Health Education and Culture, GESEC, University of Lleida, Lleida, Spain; 3 Health Care Research Group, GRECS, Biomedical Research Institute of Lleida, IRBLleida, Lleida, Spain; 4 Institut Català de la Salut and Department of Medicine - University of Lleida, Lleida, Spain

**Keywords:** Qualitative research, Primary health care, Low Back pain, Nursing, Medicine, Pain management

## Abstract

**Aim:**

To identify misbeliefs about the origin and meaning of non-specific chronic low back pain and to examine attitudes towards treatment by primary health care providers.

**Design:**

Generic qualitative study.

**Methods:**

Ten semi-structured interviews were conducted between October and November 2016 with physicians and nurses from primary health care centres in Lleida. The interviews were transcribed and analysed using inductive thematic analysis via Atlas.ti-8 software.

**Results:**

Five themes were identified: i. beliefs about the origin and meaning of chronic low back pain, ii. psychosocial aspects of pain modulators, iii. Therapeutic exercise as a treatment for chronic low back pain, iv. biomedical attitudes of primary health care providers, and v. difficulties in the clinical approach to chronic low back pain.

**Conclusion:**

Primary health care providers have a unifactorial view of chronic low back pain and base their approach on the biomedical model. Professionals attribute chronic low back pain to structural alterations in the lumbar spine while psychosocial factors are only recognized as pain modulators. For professionals, therapeutic exercise represents a possible solution to chronic low back pain; however, they still do not prescribe it and continue to educate on postural hygiene and recommend limiting physical and/or occupational activities, as opposed to clinical practice guidelines. These findings suggest that to improve the adherence of primary health care providers to the biopsychosocial model, it may be necessary first to modify their misbeliefs about non-specific chronic low back pain by increasing their knowledge on pain neurophysiology.

**Trial registration:**

ClinicalTrials.gov Identifier: NCT02962817. Date of registration: 11/11/2016.

**Supplementary Information:**

The online version contains supplementary material available at 10.1186/s12875-021-01617-3.

## Background

In Spain, chronic low back pain (CLBP) is the second most frequent chronic health problem; it has a prevalence of 18.5% [1] and accounts for up to 10–20% of primary health care (PHC) consultations [2]. In 2018, 2.6 million consultations for low back pain were attended in PHC, indicating that PHC providers (PHCPs) play an essential role in addressing this health problem [3]. In 80–90% of cases, CLBP is diagnosed by PHCPs as CLBP of non-specific origin since it is not possible to identify the cause of the pain [4, 5]. Traditionally, treatment of CLBP has focused on analgesia and activity restriction. However, this treatment is ineffective and can even be harmful [6], generating distrust among patients and frustration towards PHCPs [7]. As a support tool, PHCPs have at their access the Clinical Practice Guideline of the Catalan Institute of Health for adult lumbar spine pathology, which provides a set of recommendations aimed at improving the care of patients with CLBP [8]. Current clinical recommendations include the use of a biopsychosocial conceptual framework for the treatment of CLBP, which supports the use of education, resumption of daily activities, and therapeutic exercise (TE) but places less emphasis on pharmacological and surgical treatment and does not recommend the routine use of complementary tests [9–11].

The biopsychosocial model recognizes the influence of psychological and social factors on the perception and behaviour of pain and the development of disability. For this reason, this model has been considered more appropriate than the traditional biomedical model for understanding the multifactorial nature of CLBP and meeting the needs of patients [7]. However, PHCPs continue to address CLBP under a biomedical approach in which pain is attributed to a structural or biomechanical deficit, and treatment is focused on addressing the physical pathology and symptoms [12].

PHCP beliefs and attitudes about health and disease play a key role in the model they adopt for treating CLBP. Biomedical training in PHCPs has indirectly increased misbeliefs and attitudes about CLBP, while biopsychosocial training may decrease them [13]. Beliefs have been described as “*a cognitive process that results in concrete knowledge of how I think things are*” [14] while the term misbelief has been defined as “*a false belief, or at least a belief that is not correct in all respects*”, based on incomplete or inaccurate information [15]**.** On the other hand, attitudes are “*a more complex cognitive state that involves beliefs and feelings, as well as values and predispositions to act in a certain way*” [14]. There is evidence that PHCPs under the biomedical model or with strong fear-avoidance beliefs tend to advise patients to limit their activities [16], and may introduce or reinforce in their patients misbeliefs about CLBP and fear-avoidance behaviours that exacerbate pain and predispose to the development of disability [17–19].

Several studies have focused on understanding beliefs and attitudes about CLBP primarily among physicians and physical therapists in different countries [2, 14, 19–21]. As far as we know, PHCP beliefs and attitudes have not been studied in Spain, so it seems necessary to explore them. A greater understanding of the beliefs and attitudes among Spanish PHCPs can help to develop educational interventions to reconceptualize beliefs about the origin and meaning of pain for PHCPs, and thus to facilitate a more effective application of the biopsychosocial model in the approach to CLBP in PHC.

The research question for this qualitative study was formulated following the acronym SPICE (Setting, Perspective, Intervention, Comparison and Evaluation) [22] and is as follows:

- What are the misbeliefs about the origin and meaning of non-specific CLBP and the attitudes towards treatment of PHCP?

## Methods

### Aim

The aim of this study was to identify misbeliefs about the origin and meaning of non-specific CLBP and attitudes towards treatment by PHCPs.

### Design

This study is framed within the constructivist inquiry paradigm with a theoretical-methodological approach that is also constructivist in this case [23]. Trial registration: ClinicalTrials.gov Identifier: NCT02962817. Date of registration: 11/11/2016.

A generic qualitative design was used, which is defined as one that is not guided by an explicit or established set of philosophical assumptions, such as grounded theory, phenomenology, or ethnography [24].

### Sample

Non-probability purposive sampling was used to recruit a sample of PHC physicians and nurses. The principal investigator recruited and interviewed participants according to gender, age and health discipline to ensure maximum variation in the sample [25–27].

The inclusion criteria for PHCPs were for physicians and nurses practicing at any PHC centre who consented to participate in the study voluntarily.

The principal investigator carried out the recruitment process in two PHC centres in Lleida (Spain). The study began with a presentation face-to-face to the respective teams to inform and to solicit their involvement. Importantly, the principal investigator had no previous involvement with participants, so she introduced herself to participants as a physiotherapist specialising in neuroscience and a PhD student studying the misbeliefs about CLBP pain and the attitudes of PHCPs working in PHC centers.

A total of four participants refused to participate because of the high workload in their schedules.

The sample size was established by the thematic saturation of the results, which means until relevant information was obtained from the new participants to generate more topics [28, 29].

### Data collection

Data collection was conducted during October and November 2016 using semi-structured interviewing as the technique. Two interview scripts (physicians and nurses) (Appendix 1 and 2) were designed to collect information on PHCP beliefs and attitudes about non-specific CLBP [25]. The principal investigator (EGM) conducted the semi-structured interviews at the same PHC centres where the PHCPs work. The questions were asked in Spanish or Catalan (depending on the interviewee’s mother tongue) in an open format. All interviews were audio-recorded with the informed consent of the participants, with an average duration of approximately 30 min, and then transcribed textually for analysis. The principal investigator also resorted to note-taking during and after the interviews, which helped to interpret and to understand the discourses during the analysis.

### Data analysis

The principal investigator conducted an inductive thematic analysis to identify, to analyse, and to report issues within the data [30]. The transcripts were reviewed and imported into the Atlas.ti 8.0 software. Coding was used to code the PHCPs’ own words and compare them to develop conceptual themes. Codes were created from the interview script (deductive coding). Subsequently, the interview was rigorously read, and each sentence or paragraph with the same meaning was assigned a code from the previous list, or a new one was created if necessary (inductive coding). Finally, the codes were grouped into main themes and subthemes.

### Rigour

The participation of an external coder, who independently coded and classified the material for peer review, guaranteed the rigor and reliability of the content during the thematic analysis [31]. This study was also conducted and reported following the Consolidated Criteria for Qualitative Research Reporting (COREQ) [32].

## Results

### Description of participants

A total of ten participants was included, five physicians (two men and three women) and five nurses (four women and one man), ranging in age from 40 to 63 years (Table 1). All participants provided their written consent to participate and to record the interviews after being informed about the objectives of the study.

**Table 1:** Socio-demographic data of primary health care providers

ID Code	Gender	Age	Occupation	PCHP experience (years)	Low back pain
BM 1	Male	51	Physician	25	Yes
BM 2	Woman	47	Physician	20	Yes
BM 3	Male	63	Physician	35	Yes
BM 4	Woman	53	Physician	23	Yes
BM 5	Woman	57	Physician	25	No
RN 1	Woman	59	Nurse	36	Yes
RN 2	Woman	40	Nurse	14	No
RN 3	Woman	48	Nurse	15	No
RN 4	Woman	51	Nurse	20	Yes
RN 5	Male	53	Nurse	24	Yes

*ID* Identification, *BM* Bachelor of Medicine, *RN* Registered Nurse, *PCHP* Primary Care Healthcare Professional

### Qualitative data

Five main themes and 13 subthemes were identified (Table 2).

**Table 2:** Themes and subthemes of primary health care provider interviews

Themes	Subthemes		
**Beliefs about the origin and meaning of chronic low back pain**	Structural alterations in the lumbar spine as a cause of chronic low back pain	The influence of providers’ own low back pain experiences on their beliefs	
**Psychosocial aspects as pain modulators**	The relationship between perceived pain intensity and mood	The influence of the environment on pain	
**Therapeutic exercise as a treatment for chronic low back pain**	Therapeutic exercise improves pain, while unsupervised physical exercise aggravates the pain	Mechanisms underlying the benefits of therapeutic exercise on pain	Difficulties in prescribing therapeutic exercise
**The biomedical attitude of primary care providers**	The search for a diagnosis to justify pain	Education in postural hygiene	Recommendations for limiting work activity
**Difficulties in the clinical approach to chronic low back pain**	Primary health care providers versus pharmacological treatment of chronic low back pain	Barriers in primary health care provider-patient communication	The need for primary health care providers to acquire new knowledge about pain

#### Theme 1: beliefs about the origin and meaning of chronic low back pain

All of the support quotes extracted for topic 1 are available in Table 3.

**Table 3:** Statements by primary health care providers on beliefs about the origin and meaning of chronic low back pain

Subthemes	Quotations
**Structural alterations in the lumbar spine as a cause of chronic low back pain**	“Usually, a process that goes from effort to the degenerative process proper to age [...] An effort plus a degenerative pathology. You do not have these pains when you are young” (BM 3) “There is a physiological component that would be a lumbar hernia and a psychosomatic component” (BM 2). “I think it ends up becoming chronic because the cause that starts it is not resolved at the bottom and it is not something organic, but there are probably external things [...] Anxiety, stress, depression basically, problems, grief, loss of a loved one” (BM 4).
**The influence of providers’ own low back pain experiences on their beliefs**	“I think there is a genetic origin since my father already had a couple of hernias [...] My sister also has one” (RN 5). “I haven’t even had an MRI, so I don’t know if I have a disk disease. But I don’t want to have one, precisely for that reason, because I think that if I see a herniated disk, I will still think that it is the herniated disk. So, I prefer not to know what I have. I do not look at it so as not to change my attitude [...] I find myself more limited. I feel more insecure” (BM 4).

### Structural alterations in the lumbar spine as a cause of chronic low back pain

*"Because they already have a bad structure, spinal deviations, arthrosis problems, they have things at a structural level and then that combined with what they can do wrong, exercise, at work. Bad posture all day [...] It may be that the back pain is not specifically from the herniated disc. But normally it has started like this, with a lumbar pain and then a hernia evolves" (BM 5).*

The PHCPs in this study establish a dissociation between organic and psychological in CLBP. The PHCPs believe that when there is pain in the lumbar region, there is a structural alteration at the local level that causes it. For the PHCPs, these structural alterations in the lumbar spine, whether as a result of congenital deformities or age, combined with poor postural hygiene during physical and/or occupational activities or with physical exertion, are the cause of CLBP. The PHCPs interviewed also link age and the ageing process, to CLBP in the belief that age negatively influences the structures of the lower back region, triggering pain. In addition, they believe that pain can later trigger an injury to the lumbar spine, for example, a herniated disc. However, the PHCPs in this study also believe that psychological factors, such as stress, anxiety, and/or depression can perpetuate CLBP, but they do not recognize that stress itself is a mechanism that can produce an organic response linked to the perception of pain. The PHCPs perceive pain and psychological factors in a unidirectional way; pain carries a great psychological burden.

### The influence of providers’ own low back pain experiences on their beliefs

*"In my case, it is a herniated disk, because I have had an MRI, the chronic one can be postural too [...] Chronic means forever. I do not want to think that the pain is chronic, because if it is not very heartbreaking” (RN 1).*

Many of the PHCPs interviewed in this study admit to having suffered low back pain episodes throughout their lives. They are personal perspectives, and even family experiences influence the meaning they attribute to the pain. This meaning is based on the beliefs they hold as true about the origin of the pain.

In this study, the PHCPs with CLBP reinforce their belief that pain is associated with structural damage when using the findings observed in imaging tests to explain their pain. For the PHCPs, pain is a wrenching experience, so they refuse to accept the disease’s chronicity. Some the PHCPs also believe that CLBP has a genetic origin, influenced by family experiences, so they establish a strong association between genetics and the risk of developing herniated discs.

The PHCPs interviewed with CLBP admit to being more reluctant to undergo image tests since knowing their possible results could trigger behavioural changes because of fear-avoidance beliefs about physical exercise and/or work activity. For the PHCPs, CLBP is related to the appearance of functional limitations.

#### Theme 2: psychosocial aspects as pain modulators

All of the support quotes extracted for topic 2 are available in Table 4.

**Table 4:** Statements by primary health care providers on psychosocial aspects as pain modulators

Subthemes	Quotations
**The relationship between perceived pain intensity and mood**	“With the same pathology, there is a lot of difference with a person with a depressed mood than with a good mood. I think it modulates the pain very much, at least the perception that the person has” (BM 1). “A person who is positive, who is dynamic and who has things to do, greatly increases the pain threshold. And another one who is at home watching TV [...] Well, this one is going to notice the pain a lot more” (BM 3). “Avoid stress, avoid all those conditions that are harmful or that you perceive as harmful [...] At times when more pain appears, have resources, drugs, whatever, to control those moments of increased pain” (BM 4). “One will not endure one thing, and you see another (patient) who has the same thing and that one is tolerating it. He complains in another way. I do not know. It depends on the personalities of the patients [...] Because it gives you a psychic discomfort that worsens the physical one” (BM 5). “I don’t know exactly. I do not know if it has to do with endorphins or what. But that the pain threshold is directly related to mood, I’m sure” (BM 4).
**The influence of the environment on pain**	“People who are working because they come for lumbago from time to time. When they retire, they are invalidated, they stop working or anything else because you have them more often because of lumbago [...] because as me have nothing else to do, I’m going to see the doctor to see what this is” (BM 3).

### The relationship between perceived pain intensity and mood

*"There are people who are very limited with daily life tasks. Very disabled. So, this is linked to serious depressive conditions. Depressives pictures because they feel useless, they see that the pain does not make them rest, the character is irritable, they do not sleep at night. Well, everything is a fish that bites its own tail [...] The weaker I am, the more depressive I am, because the pain becomes much more intense. If you are an active person, optimistic, that is all there is to it, and I will pull the plug. Then you are not obsessed with pain. Character has long been very much influenced by pain, how to tolerate or not tolerate pain anymore" (RN 2).*

The PHCPs in this study believe that the patient’s mood influences his or her perception of pain, so that a low mood has a negative influence on pain, while patients who remain active and have a positive attitude have better tolerance and greater control over their pain because they pay less attention to it. As a result, the PHCPs emphasize the need for patients with CLBP to lead active lives, to avoid stressful situations, and pharmacologically to manage pain during episodes of exacerbation.

The PHCPs interviewed, in turn, also believe that disabling pain can trigger stress and mood disorders, such as depression, sleep problems, and mood swings in the patient. For the PHCPs, pain and depression create a vicious cycle in which pain produces a low mood and a feeling of sadness, and as a result, this depressive condition aggravates the pain. In addition, the PHCPs believe that this vicious circle is influenced by each patient’s personality traits and perception and/or tolerance of pain. For the PHCPs in this study, pessimistic patients with a depressive personality have greater perception and sensitivity to pain and, therefore, lower tolerance. However, the PHCPs recognize that they do not know exactly why the mood is directly related to the pain threshold. While some of the PHCPs believe it may be due to the release of endorphins, other PHCPs believe it is due to the psychological impact of emotional distress on pain.

### The influence of the environment on pain

*"I believe that there are some important psychosocial factors, the average age of life from 50 and so, that the person has already raised the children and begins to have ailments of the locomotive system, the nest begins to feel empty, because depression, sociofamily problems and that I believe is important. Also, sociability. A person who does not relate to anyone is not the same as another person who is here, there, and well has some problem [...] I think that sociability does a lot in everything” (RN 5).*

For the PHCPs, various factors, such as family environment and level of sociability, influence the perception of pain. The PHCPs emphasize the importance of interpersonal relationships in paying less attention to pain and decreasing its intensity. In addition, they highlight the work situation as another factor that influences the course and perception of pain. For the PHCPs in this study, patients who are unemployed, retired, or disabled attend primary health care consultations more frequently.

#### Theme 3: therapeutic exercise as a treatment for chronic low back pain

All of the support quotes extracted for topic 3 are available in Table 5.

**Table 5:** Statements from primary health care providers about therapeutic exercise as a treatment for chronic low back pain

Subthemes	Quotations
**Therapeutic exercise improves pain while unsupervised physical exercise aggravates the pain**	“I think it has positive repercussions if you do directed physical activity. I think it is very positive. For me the most. Then there are the negative repercussions of doing the opposite” (BM 1). “Exercise without effort, that is to say, don’t go to the gym to do weights or rowing or anything else that can make you more tired” (BM 3) “If we move, if we walk, if we try positional education of sitting, of standing, of lifting, of all this, I think it helps. Because you will not get hurt. We will not hurt our nerves or our backs” (RN 4). “Poor movement can worsen, but exercise improves pain” (BM 5).
**Mechanisms underlying the benefits of therapeutic exercise on pain**	“For the unblocking of the muscles or the strengthening of the blood supply. When one moves an area because it provides more irrigation, that area is more nourished” (RN 5). “The more rehabilitation, the more movement, because the head is also doing well. So, if you are very sedentary and very inactive, it makes your head think only of pain, sorrow, sickness, and you have more and more. If your head is very inactive and you exercise a lot, the pain is reduced” (RN 2). “We are happier (if we exercise). More well-being and more happiness” (RN 4).
**Difficulties in prescribing therapeutic exercise**	“There are patients who do not walk, you tell them to stretch or go to the physical therapist, they don’t do it [...] They prefer to take medication” (RN 4). “Do a controlled, healthy, very conscious activity [...] always supervise. For example, by a physical therapist. I think that is more appropriate than a nurse or even a doctor” (RN 5) “In some centres in Spain, they already have a physical therapist. Here it was asked for, it is asked for. It is one of the demands that is made” (BM 3).

### Therapeutic exercise improves pain while unsupervised physical exercise aggravates the pain

*"It depends on the movement and the activity [...] I believe that walking and making a life without great physical effort in a lumbar pain is positive and what is not positives is to make efforts of loads and forced movements or forced positions" (BM 2).*

The PHCPs in this study believe that performing TE, which is adapted physical exercise that is planned and supervised by a health professional, can be beneficial in the treatment of CLBP. The PHCPs recognize that TE improves the painful experience and consider it a key element in preventing musculoskeletal and nervous system injuries.

However, the PHCPs believe that unsupervised, inadequate, and poorly executed physical exercise can be detrimental to CLBP, as they link it to increased pain intensity and a worsening of underlying pathology in patients. For example, low-impact activities that do not require great physical effort, such as walking, help combat and overcome pain, while high-intensity physical exercise that requires great muscular effort or forced postures can aggravate pain.

### Mechanisms underlying the benefits of therapeutic exercise on pain

*"Exercising and all that is feeling good [...] I'm sure endorphins go off and stuff like that, but I totally ignore it. I stayed on the citric acid cycle when I studied physiology” (BM 3).*

As mentioned above, the PHCPs identified the CLBP with the presence of structural alterations. Thus, the PHCPs explain the reduction of pain using TE by the benefits it produces on the body, highlighting the functional and blood improvements in bone support, joint congruence, and muscular components.

Moreover, the PHCPs link the realization of TE to a sense of psychological well-being and a reduction in the perception of pain. For the PHCPs in this study, these benefits may be due to the release of endorphins, which occurs at a physiological level during their performance although they acknowledge that they do not know the mechanisms underlying the effects of TE on pain.

### Difficulties in prescribing therapeutic exercise

"If we have to prescribe exercise, we almost do it from our personal experience of sport. But not because we know how to prescribe exercise" (BM 1).

The PHCPs in this study believe that patients with CLBP would improve with the prescription of TE, as they believe that leading a sedentary lifestyle predisposes them to paying greater attention to pain, thereby increasing the intensity of perceived pain. On the other hand, since they cannot refer their patients to the physical therapist and do not know the parameters that make up the correct TE prescription, they provide recommendations from their personal and clinical experience. However, the PHCPs interviewed also recognize that some patients do not follow the recommendations they provide to stay active, as they prefer to receive pharmacological treatment to alleviate their chronic pain. Thus, the low therapeutic adherence showed by some patients with CLBP also contributes to the low level of TE prescription by the PHCPs.

The PHCPs insist on the need to incorporate TE into the treatment plan of patients with CLBP, although they consider that the prescription and supervision of TE should be carried out by a trained health professional. For the PHCPs, the physiotherapist is the PHCP who should prescribe ET to patients with CLBP. The PHCPs believe that physiotherapists can help control the patients’ pain and/or speed up their recovery, which is why they demand their incorporation into primary health care teams.

#### Theme 4: the biomedical attitude of primary health care providers

All of the support quotes extracted for topic 4 are available in Table 6.

**Table 6:** Statements on the biomedical attitude of primary health care providers

Subthemes	Quotations
**The search for a diagnosis to justify the pain**	“It has several causes [...] It can be degeneration, bad posture, accident, psychological [...] People don’t know where the pain comes from and you can’t explain it to them either” (RN 3) “There are patients who ask for imaging tests and, sometimes, doctors in front of the pressure of the user or his relative have asked for a radiological test without it being indicated” (BM 1)
**Education in postural hygiene**	“The head of the rehabilitation service passed us some sheets [...] when I see that it is a positional, an overload, well I tell him, do these exercises [...] read it and such. I’ll explain the two or three most important ones” (BM 2).
**Recommendations for limiting work activity**	“Rest when they are in acute pain, relative rest, because rest is not at all” (RN 3). “I follow my recommendations. I do not even remember. I am sure I have read it and will follow it, but now I do not remember [...] knowing them. What happens is that I assume part of the guide and then the other is my day to day” (BM 2). “Evidence-based medicine, but the one the patient explains to you [...] Maybe it’s just to help the patient, but maybe it’s just to get the patient off our backs, I don’t know. There is a mixture of things [...] Many times even the advice we give is wrong” (BM 4) “I am a professional who treats chronic low back pain and at the same time I am a user of chronic low back pain. It is not a clinical guide, but it is a direct experience” (RN 5).

### The search for a diagnosis to justify the pain

*"In most patients you receive you end up finding a cause or you end up detecting something because you want to give the patient the explanation that that is the cause. It's easy for you to say: it's that you're in pain because you have a hernia [...] I guess it's easy for us to explain it that the way and it's also easier for the patient to understand, because having pain without a cause is hard" (BM 4).*

The PHCPs identified a unifactorial view of pain. However, for them, there are many possible causes of CLBP, both organic and psychic, so they recognize that they have difficulty explaining the origin of pain to their patients. Sometimes the PHCPs feel pressured by their patients and/or family members to find a cause for the pain. This situation forces the PHCPs to request imaging tests that are not indicated.

The responsibility of the PHCPs to identify the cause of the pain means that the PHCPs will eventually use the results of the imaging tests to explain the CLBP to their patients. It is easier for professionals to explain pain to their patients when they have found a structural cause to which they can attribute it, and they believe that, in this way, patients will understand it more easily.

### Education in postural hygiene

*"We insist much on postural education, on effort, on weight, on not being too much immobile” (RN 4)*

The PHCPs in this study provide during their consultations specific recommendations on postural hygiene to patients, based on educational material developed by the Rehabilitation Service, for the prevention and/or control of CLBP. The PHCPs educate their patients on the best posture to adopt, either during physical exercise or in the workplace. Sometimes, when they suspect that the cause of the pain could be bad posture and/or muscle overload, they also provide their patients with a sheet of reinforcement exercises to insist on the notion of maintaining correct postural hygiene to prevent a new episode of acute pain. However, the PHCPs recognize that they do not spend enough time in their consultations to explain all the exercises provided to their patients.

### Recommendations for limiting work activity

*"Let them change jobs, because if they work in construction carrying weight, it's certainly not the best job for a chronic low back pain. Let them look for more sedentary jobs" (BM 2).*

For the PHCPs, acute low back pain corresponds to periods when patients have had a higher level of activity. For this reason, during acute pain episodes, they recommend that their patients reduce their work activity when it requires great physical effort. In addition, they recommend that patients change jobs when they believe that the mechanical factors inherent to the work activity cause the establishment and perpetuation of the pain.

The PHCPs in this study believe that patients with CLBP should lead a sedentary life to prevent a new episode of acute pain although the Clinical Practice Guidelines of the Catalan Institute of Health for adult lumbar spine pathology emphasise the need for patients with CLBP to remain active. However, the PHCPs recognize that they do not follow the recommendations offered in that guide and provide their patients with CLBP with recommendations based on their clinical and personal experience; however, they believe that this approach may be counterproductive in some cases.

#### Theme 5: difficulties in the clinical approach to chronic low back pain

All of the support quotes extracted for topic 5 are available in Table 7.

**Table 7:** Statements by primary health care providers about the difficulties in the clinical approach to chronic low back pain

Subthemes	Quotations
**Primary health care providers versus pharmacological treatment of chronic low back pain**	“Mainly NSAIDs, non-steroidal anti-inflammatory drugs, and depending on age, either paracetamol or diclofenac or ibuprofen. And then, because later they would be plotted and then if there was root affectation with paraesthesia or neuralgia or something, then we would give the gabapentin” (BM 3) “People seek immediacy, the immediate solution, they have a false expectation of medicine. There are many factors that influence them, and they do not accept them” (BM 2) “The pharmacological route is very powerful. Today no one has had pain, no one. Furthermore, I believe that it is considered medical malpractice for a patient to have pain” (RN 5) “We give him little solution and as a doctor you feel a little helpless [...] I think there would surely be other professionals more suited to treat him. More effectively at least” (BM 1) “If he doesn’t get better I’d rather have him treated by another professional because I do not offer him anything” (BM 5). “Poor pain control and if I see something in the complementary tests, since what we were saying is a disk disease, which you can see is compressive, then I also make a referral [...] When I send him to rehab it is because they usually make them a school of the back to work on postures, like sitting, like taking weights [...] I don’t think they get better. I think they are given instruments, let’s say, to help them live with the pain” (BM 4). “The patient just complains. That I am in pain, that I have functional impotence that, I am in pain. So, if I’ve finished the therapeutic arsenal and I do not see anything that can be done, then I’ll send you to the orthopaedic surgeon [...] At least you can be sure that the specialist already agrees with you” (BM 3).
**Barriers in primary health care provider-patient communication**	“Everyone’s pain threshold is very questionable. But of course, if they tell you that they have a pain because you eradicate it, you go towards that pain at the level of the lower back” (RN 2). “Real, for them, I guess. The thing is that it’s subjective, because sometimes I think of a person who comes in and tells me a pain that maybe for him is very intense and I think that maybe it’s less” (BM 5). “They come to you at sixty years old or sixty-something who are waiting to retire and then retire at once [...] And then you never know if they have the pain or increase it looking for some benefit, because I want a disability” (BM 3) “In our practice we have to treat the pain of that person, the analysis, hypertension... Then, the pain will not kill him because there it is. We try to focus it, treat it and help it. But let’s say that it also surpasses us a little [...] probably what the patient is most concerned about is the pain, but probably what I am least concerned about is the pain. Although I understand that it affects his quality of life a lot” (BM 4). “A pain of chronic characteristics will not improve in three days with the treatment we do either” (BM 1). “Always all the education we give our patients about any health problem when they leave, first of all they will get half or less of what I say, because people are blocked, you are in a consultation and then they are telling you that you have lumbago [...] that this can happen to you, that the other can happen to you, that treatment is here and there, that this is forever, that this has no cure, that this is a life sentence. So, all that information that reaches the patient is blocked, and then it goes away. Of what you have told him, what does he get, 40%? Of that 40, 20% is agreed upon, and so at the end, a residual 10% remains as a reminder of the interview you had with him” (BM 3).
**The need for primary health care providers to acquire new knowledge about pain**	“From the primary school management, they offer specific training in pain management. But the truth is that the approach is always pharmacological [...] well, of course, we do have pharmacological knowledge and the latest developments in drugs as well, but little else” (BM 1). “How can we approach that chronic patient who has taken everything? How can I approach him to control all the symptoms a little? Not only medication, a more complete, more global approach [...] Alternative things to pharmacology, rehabilitation. Different alternative things. Even on a psychological level” (BM 2). “I’d like to be given the keys to dealing with it successfully, basically” (BM 4).

### Primary health care providers versus pharmacological treatment of chronic low back pain

*"There are patients in whom in the pain (the drug) works, but there are many patients, especially in the chronic one that does not work. Then, you really feel very helpless with the drugs [...] Probably because there are many emotional causes there, so what you do is have these patients consult and re-consult" (BM 4).*

For the PHCPs in this study, drug treatment is the first line of treatment for CLBP. The most commonly prescribed drugs are low-dose non-steroidal anti-inflammatory drugs (ibuprofen, naproxen) and/or paracetamol, the latter sometimes combined with tramadol. In addition, the PHCPs add gabapentin when there is evidence of root involvement. However, they recognize that drugs are not always effective in treating and controlling CLBP.

The PHCPs interviewed consider CLBP a frequent reason for consultation that is very difficult to treat since, on many occasions, pharmacological treatment is not very effective. The PHCPs believe that pharmacological failure cases are due to the influence of emotional factors associated with pain. However, they also believe that patients do not accept the affective and cognitive dimensions of the painful experience, so they repeatedly come for a consultation in search of a solution to their pain. However, for the PHCPs, the battery of drugs they can prescribe to this type of patient is minimal, and they believe that they have no other therapeutic tool other than pharmacology to achieve adequate pain management.

The PHCPs in this study feel frustrated and powerless in the face of the low efficacy of the drugs they prescribe. The generally poor results obtained with the analgesic treatment make CLBP a “bad treatment” pathology since they believe that the pharmacological route is powerful and that no patient should have pain. Further, the PHCPs believe that providing an ineffective treatment is medical malpractice. Consequently, when the CLBP does not improve with drug treatment, the PHCPs end up resigning themselves and referring their patients for treatment by a specialist health professional.

For the PHCPs interviewed, the referral of patients to other health professionals is an opportunity to verify and/or to agree on their diagnosis and treatment and, therefore, to address the CLBP approach with greater certainty in the face of the low efficacy of drug treatment. For this reason, the PHCPs recognize that, given the persistence of pain, its poor analgesic control, the presence of structural alterations found in imaging tests, and the repeated complaints of the patient in medical consultations, they end up referring patients with CLBP to the Trauma Service or the Rehabilitation Service to provide them with guidelines on postural hygiene and ergonomics. However, the PHCPs refer their patients to the Rehabilitation Service, even though they believe they will not improve.

### Barriers in primary health care provider-patient communication

*"You always have to believe that the person is in pain [...] You always have to treat them as if they are. But a lot of times you think, he's not in pain, or he's in pain like that and he's telling me he's in pain" (RN 3).*

Pain is a physical and emotional barrier to primary health care provider-patient communication. For the PHCPs in this study, CLBP is challenging to diagnose and to treat because of the pain intensity’s subjectivity. While some PHCPs accept the subjectivity of pain and address it without question, other PHCPs question their patients’ pain, even if they end up treating it.

The PHCPs interviewed believe that some patients somatise or verbalize a greater intensity of pain when emotional or economic interests are related to their pain, such as sick leave, retirement, or compensation. As a result of these beliefs, the PHCPs do not validate pain; that is, they do not recognize their patients’ pain as real or show empathy.

The PHCPs in this study are least concerned about their patients’ CLBP, believing that chronic pain will not get better and that there is nothing they can do about it. However, the PHCPs also believe that patients do not want to have pain and expect a quick and effective solution to their CLBP from their physician’s appointments. Nevertheless, the PHCPs recognize that the consultation time they have for each patient is insufficient to address all comorbidities in addition to the CLBP. Consequently, the explanations provided by the PHCPs for CLBP are brief and based on the identification of diagnosis, treatment, and prognosis, with emphasis on the chronicity of the pain. In addition, the PHCPs do not check patients’ understanding of the explanations provided and recognize that a lack of PHCP-patient understanding contributes to patients not remembering the information provided in consultations. The PHCPs interviewed feel that CLBP’s clinical approach is beyond them.

### The need for primary health care providers to acquire new knowledge about pain

*"There are the nociceptive, proprioceptive pains, all those sensitivities that will have something to do with it. I do not know, I do not know, that I'm a general practitioner [...] Knowledge doesn't occupy a place. It takes time [...] Keep in mind that we are not capable of covering everything we need of everything; we keep the 4 most basic things and that's it" (BM 3).*

As mentioned above, the PHCPs provide CLBP patients with recommendations based on their clinical and personal experience. This approach leads the PHCPs to recognize that their knowledge of the mechanisms underlying CLBP is limited and to justify their lack of knowledge by believing that, as PHCPs, they cannot fully understand and address all conditions during their consultations. Instead, the PHCPs in this study believe they have all the pharmacological knowledge necessary to treat patients with CLBP, even though, in most cases, drug treatment is ineffective. For this reason, the PHCPs want to know about non-pharmacological therapeutic alternatives to improve and/or to control CLBP, including addressing the psychosocial aspects associated with pain. The PHCPs want to know how to carry out effective and comprehensive management of CLBP.

## Discussion

This study aimed to identify misbeliefs about the origin and meaning of non-specific CLBP and attitudes towards treatment by PHCP. Our results suggest that the PHCPs have a unifactorial view of CLBP and that they base their approach on the biomedical model. This study reveals several controversies in the PHCPs during the management of CLBP. We found that for the PHCPs, the cause of CLBP is structural alterations in the lumbar spine and that psychosocial factors can modulate and perpetuate pain. However, they do not recognize that psychosocial factors are themselves a possible trigger. Although the PHCPs see TE as a possible solution to CLBP, they still do not prescribe it and continue to educate on postural hygiene and recommend limiting physical and/or occupational activities, as opposed to clinical practice guidelines and scientific evidence. The PHCPs question their patients’ pain, even when they request imaging tests, even when they are not indicated, and end up treating it. Given the low effectiveness of drug treatment, the PHCPs in this study refer patients to specialized care teams, knowing that their patients will not improve.

The PHCPs attribute the CLBP to structural alterations in the lumbar spine that are characteristic of the ageing process. According to our results, Sit et al. [2] demonstrated the presence of biomedical beliefs about the origin and significance of CLBP in PHC physicians in Asia. However, to our knowledge, the influence that personal experiences have on PHCP beliefs has not been studied. Our results have shown that the PHCPs in this study have developed a relationship between their personal experiences of CLBP and their biomedical beliefs.

One of the main findings of this study is that the PHCPs in this study believe that stress, anxiety, or depression can perpetuate CLBP. The PHCPs also believe that the patient’s mood and family environment, and level of socialization influence CLBP although they acknowledge that they lack sufficient knowledge of the neurophysiology of pain to explain the relationship between the painful experience and psychosocial factors [33]. There is abundant scientific evidence supporting that psychosocial factors play a more critical role than biomechanical factors in developing and perpetuating chronic pain [34]. However, our results suggest that PSAPs base their clinical practice on strictly biomedical training focused on identifying and fixing a presumptive pathoanatomical cause, which exacerbates misbeliefs about the origin of CLBP and moves them away from the adoption of the biopsychosocial model in CLBP management, as recommended by current Clinical Practice Guidelines [35].

Our results show that the PHCPs rely more on their clinical and personal experience than on clinical practice guidelines, which contain a set of recommendations aimed at improving the care of patients suffering from low back pain. This suggests that the PHCPs show low adherence to the Clinical Practice Guidelines, facilitating their expertise during the management of CLBP. Adoption of low back pain guidelines is a well-documented problem. Slade et al. [36] showed that PHC physicians were more confident in their clinical experience because they believed that clinical guidelines for the management of low back pain lacked credibility, were prescriptive and hindered clinical reasoning or professional autonomy [36]. More recently, Hall et al. [37] showed that physicians face numerous barriers to providing evidence-based LBP care particularly regarding appropriate use of imaging, use of simple analgesics versus opioids for pain relief, and providing advice to stay active.

The PHCPs in this study believe that TE can be beneficial in the treatment of CLBP but do not prescribe it because they believe that unsupervised, inadequate, and poorly executed physical exercise can aggravate pain. However, ET has been shown to have few adverse effects and to improve chronic pain severity, functionality, and quality of life [38–40]. Furthermore, according to the findings of Kolber, et al. [41], ET was the only intervention that provided sustained benefits on pain up to 48 weeks in patients with CLBP. One possible explanation for why the PHCPs see physical exercise as an aggravating factor may be in their own fear-avoidance beliefs, which decrease the likelihood that they will recommend physical exercise to their patients [16]. The PHCPs in this study, based on their biomedical beliefs, recommend in episodes of acute pain, maintaining proper postural hygiene, and limiting physical exercise and/or work activity. These results coincide with those of Darlow et al. [16], which showed that professionals with biomedical and fear-avoidance beliefs tend to limit physical exercise and work activity in patients. It has been shown that patients interpret these recommendations as requiring them to protect their backs, increasing hypervigilance and psychological distress in them [42], making it difficult for them to adhere to TE programmes [43].

Our findings also show that some PHCPs question their patients’ pain and therefore are not validating pain in their consultations, understanding this validation as a process in which the patient’s thoughts and feelings are recognized and understood, so that their absence aggravates psychological distress and negative feelings in patients [44].

Another important finding is that the PHCPs feel pressure from their patients and families to find a cause for the pain. Consequently, the PHCPs request imaging tests, even when they are not indicated, and thus attribute the pain to the structural alterations found. In line with our results, Slade et al. [36] showed that physicians were more likely to prescribe imaging tests for more demanding patients and that physicians used the findings observed in imaging tests to explain the problem and encourage optimism, under the perception that patients might doubt their clinical ability if they did not identify the cause of the pain. This biomedical approach makes analgesic drugs the first line of treatment for CLBP, although the PHCPs recognize that they are not always effective for pain. Poor analgesic pain control leads to frustration in the PHCPs. Cherkin et al. [45] showed that PHC physicians were more likely to be frustrated and less confident that their patients with CLBP were satisfied with their care because they had the perception that they were not well enough prepared to address low back pain effectively. The PHCPs perceive that patients expect medical consultations to provide a quick and effective solution to their CLBP. However, they believe that chronic pain will not improve and is not a clinical priority. Our findings are consistent with those of Sanders et al. [46], which showed that PHC physicians perceived nonspecific CLBP as a “low” health problem that they gave lower priority to, compared to other chronic diseases, because they believed that many patients did not need a physician’s consultation. In this same study, PHC physicians also emphasized the impact of patient demands on their referral decisions [46].

Similarly, the PHCPs in our study refer patients to specialty care teams for repeated complaints when pain persists, or inadequate pain control is present. Slade et al. [36] showed that physicians feel unsafe when dealing with low back pain compared to other professionals such as physical therapists. This finding is consistent with our results, which show that referral to another specialist professional gives the PHCPs greater confidence in diagnosing and treating CLBP because they believe they do not have sufficient knowledge to address CLBP from a biopsychosocial perspective.

The qualitative results of this study suggest the need for the PHCPs to receive biopsychosocial pain training since the biomedical model adopted is ineffective for the treatment of CLBP and insufficient for understanding and addressing the multifactorial nature of pain. Considering that biopsychosocial interventions with a clear focus on psychosocial factors (understanding pain, unhelpful thoughts, coping styles and goal setting) were found to be as effective as ET interventions in patients with CLBP [47], the design of biopsychosocial educational interventions is a possibility to increase knowledge in pain neurophysiology and to change PHCPs ‘misbeliefs about CLBP. The authors of this study aim to use the results of this study to develop and to evaluate, in a subsequent study, a biopsychosocial educational tool to modify misbeliefs and attitudes in PHCPs, which impact the application of a biopsychosocial care model and more effective than the biomedical model in reducing pain and disability in patients [47, 48].

### Limitations

The main limitation of this study is the date of the interviews and the number of participants, although was sufficient to reach thematic saturation and explore in depth the misbeliefs of PHCPs. In addition, the choice of purposive sampling may have been influenced by the researcher’s judgements during the recruitment of participants. Other limitations may be due to the interpretation of the data provided by the PHCPs and the local context in which the study took place.

Finally, concerning the interviews, the fact that a physiotherapist conducted the interviews could have reinforced the existing professional hierarchy or made the PHCPs feel they were being questioned or evaluated, leading to distancing behaviours that could have influenced their responses.

## Conclusion

This study shows that PHCPs have a unifactorial vision of CLBP and base their approach on the biomedical model. PHCPs attribute CLBP to structural alterations in the lumbar spine while psychosocial factors are only recognized as pain modulators. For PHCPs, the TE represents a possible solution to the management of CLBP. However, they still do not prescribe TE and instead continue to educate patients about postural hygiene and recommend limiting physical and/or occupational activities, in contrast to the Clinical Practice Guidelines. These findings reveal a gap between scientific evidence and clinical practice. Additionally, this study suggests the need to increase knowledge of the neurophysiology of pain among PHCPs, to modify erroneous beliefs about low back pain. The latter could increase the adherence of PHCPs to the biopsychosocial model and the adoption of communication techniques and strategies that improve the PHCP-patient relationship.

## Supplementary Information

**Additional file 1.**

**Additional file 2.**

## Data Availability

The datasets (content of interviews) analyzed for this study are available from the first author upon reasonable request.

## Abbreviations

CLBP: Chronic Low Back Pain
PHC: Primary Health Care
PHCPs: Primary Health Care Providers
TE: Therapeutic Exercise
